# Effect of photodynamic therapy in combination with ionizing radiation on human squamous cell carcinoma cell lines of the head and neck

**DOI:** 10.1054/bjoc.2000.1328

**Published:** 2000-08-16

**Authors:** R Allman, P Cowburn, M Mason

**Affiliations:** Research Department, Velindre Hospital, Whitchurch, Cardiff, CF14 2TL, Wales, UK; Department of Medicine, University of Wales College of Medicine, Cardiff, CF14 4XX, Wales, UK

**Keywords:** photochemotherapy, 5-aminolaevulinic acid, head and neck neoplasms, squamous cell carcinoma

## Abstract

Photodynamic therapy (PDT) is a promising treatment modality for head and neck, and other tumours, using drugs activated by light. A second generation drug, 5-aminolaevulinic acid (5-ALA), is a precursor of the active photosensitizer protoporphyrin IX (PpIX) and has fewer side-effects and much more transient phototoxicity than previous photosensitizers. We have investigated the effect of 5-ALA mediated PDT in combination with γ-irradiation on the colony forming ability of several human head and neck tumour cell lines. The effect of treatments on the DNA cell cycle kinetics was also investigated. Our results indicate that the combination of 5-ALA mediated PDT and γ-irradiation results in a level of cytotoxicity which is additive and not synergistic. 5-ALA mediated PDT had no discernible effect on DNA cell cycle distributions. γ-irradiation-induced cell cycle arrest in G2 did not enhance the phototoxicity of 5-ALA. © 2000 Cancer Research Campaign


*British Journal of Cancer* (2000) **83**(5), 655–661
© 2000 Cancer Research Campaign
doi: 10.1054/ bjoc.2000.1328, available online at http://www.idealibrary.com on IDEAL®

### Effect of photodynamic therapy in combination with ionizing radiation on human squamous cell carcinoma cell lines of the head and neck

**R Allman[1], P Cowburn[1] and M Mason[2]**

[1]Research Department, Velindre Hospital, Whitchurch, Cardiff CF14 2TL, Wales, UK, [2]Department of Medicine, University of Wales College of Medicine, Cardiff CF14 4XX, Wales, UK

**Summary** Photodynamic therapy (PDT) is a promising treatment modality for head and neck, and other tumours, using drugs activated by light. A second generation drug, 5-aminolaevulinic acid (5-ALA), is a precursor of the active photosensitizer protoporphyrin IX (PpIX) and has fewer side-effects and much more transient phototoxicity than previous photosensitizers. We have investigated the effect of 5-ALA mediated PDT in combination with γ-irradiation on the colony forming ability of several human head and neck tumour cell lines. The effect of treatments on the DNA cell cycle kinetics was also investigated. Our results indicate that the combination of 5-ALA mediated PDT and γ-irradiation results in a level of cytotoxicity which is additive and not synergistic. 5-ALA mediated PDT had no discernible effect on DNA cell cycle distributions. γ-irradiation-induced cell cycle arrest in G2 did not enhance the phototoxicity of 5-ALA. © 2000 Cancer Research Campaign

**Keywords:** photochemotherapy; 5-aminolaevulinic acid; head and neck neoplasms; squamous cell carcinoma

Photodynamic therapy (PDT) is a method for the treatment of cancer that involves the administration of systemic or topical photosensitizing drugs that are preferentially taken up by the tumour and then activated in the presence of light to cause tissue destruction (Dougherty, 1988). Photodynamic therapy works by the generation of singlet oxygen that results in damage to cell membrane structures, microvascular ischaemia, and tissue necrosis. Numerous clinical trials of photodynamic therapy have been conducted over the past decade and PDT has been approved for clinical use in recurrent bladder carcinomas, obstructing oesophageal tumours, and early carcinomas of the bladder, oesophagus, stomach, and tracheobronchial tree. PDT has also been shown to provide curative treatment of early carcinomas of the head and neck, including the oral cavity, pharynx, and larynx (Biel, 1995; Feyh, 1996). Initially PDT was tested in advanced cancers of the head and neck that were untreatable or refractory to conventional therapy, however these trials produced only limited success (Schuller et al, 1984; Wile et al, 1984). A recent retrospective review of the clinical data available for the treatment of head and neck neoplasia using photodynamic therapy indicated that complete response rates of 89.5% are achievable for early squamous cell carcinoma of the head and neck (Biel, 1998). Based on a range of photosensitizers and treatment modalities, cure rates of 95% and 80% were obtained for carcinoma in situ and T1 squamous cell carcinoma of the vocal chord and oral cavity/tongue respectively (follow up 70 months) (Biel, 1998).

*Received 21 January 2000*
*Revised 27 April 2000*
*Accepted 1 May 2000*

*Correspondence to:* R Allman

Most of the available photosensitizers, until recently, have been mixtures of porphyrins such as haematoporphyrin derivative and Photofrin (Quadralogic Technologies, Vancouver, Canada). The main problem with these first-generation photosensitizers is that of prolonged skin photosensitivity. Phototoxic incidences of 20–40% have been reported during follow-up of patients having received Photofrin, with a mean duration of skin photosensitivity exceeding 6 weeks (Dougherty et al, 1990).

The use of 5-aminolaevulinic acid (ALA) represents a different strategy in the administration of photosensitizers. ALA itself is not the photo-active drug, but rather it induces, in situ, the synthesis of a pure endogenous porphyrin called protoporphyrin IX (PpIX). The formation of PpIX forms part of the haem synthesis pathway and all nucleated cells that use oxidative metabolism are probably capable of forming this photosensitizer. However, malignant tissue appears to preferentially accumulate PpIX, forming the basis of photodynamic therapy in cancer (Battle, 1993; Kennedy and Pottier, 1994). Intravenous ALA is rapidly cleared from the body, with no PpIX fluorescence within the skin or other body organs detectable after 24 h (Kennedy et al, 1991).

Because of this reduced phototoxicity and excellent tumour localizing properties, ALA has been used with great success for the treatment of several neoplastic diseases, particularly of the skin, bladder and oral cavity. The results show very good clinical and cosmetic responses (Peng et al, 1997). However, the effects on thicker lesions (>1 mm) remain to be improved.

At present, the majority of head and neck tumours are treated conventionally with either surgery or ionizing radiation, although newer treatments are under investigation (van Dongen and Snow, 1997). Because many successful forms of cancer treatment rely upon a combination of treatment modalities, we have therefore investigated the treatment of head and neck carcinoma cell lines with various combinations of ionizing radiation and photodynamic therapy following administration of 5-aminolaevulinic acid.

### MATERIALS AND METHODS

### Cell lines and culture conditions

The three tumour cell lines used in this study; V134, V175, and SCC-61, are all human squamous cell carcinoma cell lines derived from tumours of the head and neck. The SCC-61 cell line, kindly donated by Dr AC Begg (Amsterdam, The Netherlands), has been previously described (Weichselbaum et al, 1984, 1988). The V134 (Champion et al, 1995) and V175 (Champion et al, 1997) cell lines were both derived at Velindre Hospital from patient tumour biopsy samples. Tumour cells were maintained as monolayer cultures in Dulbecco's Modified Eagles Medium (DMEM, Gibco, Paisley, UK) containing 10% fetal calf serum (Gibco, Paisley, UK), 100 U/ml penicillin (Sigma, Poole, UK 100 μg/ml streptomycin (Sigma, Poole, UK), and 0.4 μg/ml hydrocortisone (Sigma, Poole, UK).

Normal human fibroblasts were derived from skin biopsy material obtained during breast reduction surgery of non-cancer patients. Fibroblasts were maintained as monolayer cultures in DMEM containing 15% fetal calf serum, 100 U/ml penicillin, and 100 μg/ml streptomycin.

All cells were cultured at 37°C in a humidified atmosphere of 5% $CO_2$ in air. Cells were routinely subcultured by the addition of 0.02% trypsin/0.05% EDTA (Sigma, Poole, UK). Experiments were performed using cells harvested in log phase growth.

### Irradiation

All irradiations were performed at room temperature in oxic conditions using a $^{137}$Cs gamma source of 0.66 MeV energy. The dose rate was 1.2 Gy/min. Irradiations were usually completed within 15 min. Cells were irradiated as monolayers.

### Clonogenic cell survival

Exponentially growing cells were irradiated in 25 cm$^2$ flasks in 5 ml culture medium. Triplicate flasks were set up for non-irradiated controls and for each radiation dose and each experiment was repeated three times. Surviving cells were allowed to grow for 10–14 days prior to fixing and staining. Resultant colonies were fixed in 4% formaldehyde in PBS and were stained with a 1% solution of crystal violet. Colonies consisting of more than 50 cells were counted.

### Measurement of protoporphyrin IX accumulation

Confluent cell cultures were removed from their flasks by trypsinization and resuspended in serum supplemented medium to neutralize the trypsin. The cells were centrifuged at 150G for 5 min and the pellet resuspended in freshly prepared serum-free medium containing 1 mMol/l 5-aminolaevulinic acid (ALA, Sigma, Poole, UK) to give about $1 \times 10^6$ cells per 5 ml of medium. The cell suspensions were placed in 25 cm$^2$ flasks lined with a layer of 2% agar to prevent the cells attaching to the plastic. A paired flask of cell suspension in serum-free medium, but with no ALA was also prepared to act as a control. A 0.5 ml sample cell suspension was immediately removed from each of the paired flasks to measure the background fluorescence. Cell suspensions were incubated in the dark at 37°C in an atmosphere of 5% $CO_2$ in air. Samples were removed every hour from both sets of flasks to measure ALA-induced fluorescence. Flasks were gently shaken at these times but otherwise left undisturbed.

Cellular fluorescence was quantified with a FACScan flow cytometer (Becton-Dickinson, Oxford, UK). ALA-induced fluorescence was excited with an Argon laser emitting at 488 nm and emission was collected by a photomultiplier tube after passing through a 650 nm longpass filter. Data from $5 \times 10^3$ cells were recorded and processed using the LYSIS II software (Becton-Dickinson, Oxford, UK). Using side- and forward-scatter signals, debris was excluded from the final data. ALA-induced fluorescence was determined at various times by subtracting the fluorescence of the control cell suspensions from that of the ALA-incubated cells. The source of ALA-induced fluorescence was confirmed to be caused by the accumulation of protoporphyrin IX by checking the emission spectra of a sample of ALA-incubated cells with a standard protoporphyrin IX solution on a spectrophotometer (Perkin-Elmer, Beaconsfield, UK).

### Photodynamic therapy

Exponentially growing cells were incubated for 4 hours in serum-free medium containing 1 mM/L ALA. An incubation period of 4 hours was chosen as in vivo studies of the fluorescence kinetics of ALA uptake in an animal model suggested this to be the optimum (Loh et al, 1993) and was further confirmed by our in vitro studies described in the present report.

Serum-free medium was used because protoporphyrin IX is lipophilic and rapidly diffuses out of the cell into medium-containing serum. The pH of the ALA dissolved in medium was maintained between 7.2 and 7.6. The cells were then incubated at 37°C in 5% $CO_2$ in the dark. After 4 h of incubation, each flask was then exposed to light from a tungsten-halogen lamp (Micromark, London, UK) for a specific time. The total spectral irradiance at the level of the cells, and in the presence of a water filter, was 50 mW/cm$^2$ (400–750 nm) measured using an Ophir Nova power meter (Ophir Optronics, Jerusalem, Israel) fitted with a black-body absorber pyroelectric head. These measurements indicated that the irradiance was constant over the area occupied by the tissue culture flask. Ultra violet light was minimal and measured at <1 mW/cm$^2$ with UVA <15 μW/cm$^2$. Infrared radiation was minimised using a 3.5 cm water filter between the cells and the light source. Flasks containing medium and exposed to the water-filtered light source for 20 min did not warm by >1°C. Experiments were performed on at least three separate occasions.

### DNA contents

Cell cultures were removed from their flasks by trypsinization and resuspended in phosphate buffered saline (PBS). 125 μl of propidium iodide (0.4 mg/ml)/Triton X-100 (1% v/v) was added to 1 ml of cell suspension together with 50 μl Ribonuclease A (Sigma, Poole, UK 10 mg/ml). Cells were incubated at 37°C for 10 min prior to analysis with a FACScan flow cytometer (Becton-Dickinson, Oxford, UK). Propidium iodide fluorescence was excited with an Argon laser emitting at 488 nm and emission was collected by a photomultiplier tube after passing through a 650 nm longpass filter. Data from $5 \times 10^3$ cells were recorded and processed using the LYSIS II software (Becton-Dickinson, Oxford, UK). Using side- and forward-scatter signals, debris was excluded from the final data. Normal human lymphocytes were used to monitor instrument performance and to calibrate measurements of DNA ploidy. Where required, DNA histograms were deconvoluted according to the protocol of Watson et al (1987) and

© 2000 Cancer Research Campaign

Ormerod et al (1987), allowing accurate calculations of the number of cells in each of the cell cycle phases (G1, S, G2) to be made.

### Clonogenic cell survival following combined PDT and γ-irradiation

Exponentially growing cells were irradiated in 25 $cm^2$ flasks in 5 ml culture medium either 24 hours before or 24 hours following a single, fixed 'dose' of PDT. The light dose used for the PDT was 3 minutes, which from our data resulted in a surviving fraction in the region of 0.6. Triplicate flasks were set up for non-irradiated controls and for each radiation dose and each experiment was repeated three times. Surviving cells were allowed to grow for 10–14 days prior to fixing and staining. Resultant colonies were fixed in 4% formaldehyde in PBS and were stained with a 1% solution of crystal violet. Colonies consisting of more than 50 cells were counted.

### RESULTS

### Time-dependent protoporphyrin IX accumulation

All cells incubated in serum-free medium containing 1 mM/l ALA over a 24 h time period showed a time dependent increase in fluorescence. However, flow cytometry of the various cells at 22–24 h showed a wide variation in their side- and forward-scatter signals. This suggests loss of viability and some degree of cell disintegration when cells are incubated in serum-free medium for this duration together with the forced prevention of cell attachment. In the case of the VB001 normal fibroblasts almost all of the cells were fragmenting due to apoptosis following the prolonged serum deprivation. Therefore, all further PDT experiments were performed under optimal conditions of 4 h incubation in 1 mM/l ALA. The fluorescence kinetics of the individual cell lines are summarised in Figure 1.

### Clonogenic cell survival following photodynamic therapy

All of the cell lines examined had a dose-dependent clonogenic survival response to light, after 4 h of incubation in the presence of ALA (Figure 2). The data shown are from pooled experiments ($n \geq 3$). The data were fitted by a linear quadratic function for each cell line by means of a least squares Marquardt algorithm (Nash, 1979). In order to compare the responsiveness of the cell lines to ALA-mediated photodynamic therapy we used the surviving fraction following 3 min of PDT as an arbitrary value and termed this SF3. The SF3 value was derived by solving the equation $SF_3 = e(-(\alpha D + \beta D^2))$ for D = 3 min of light exposure. A fairly wide variation in PDT sensitivity was observed in these cell lines with $SF_3$ values of 0.54 (VB001), 0.67 (V134), 0.79 (V175) and 0.68 (SCC-61). The differing sensitivities were not correlated with the relative accumulation of protoporphyrin IX by the different cell lines during the 4 h incubation period.

Cells exposed to ALA but no light showed no increase in cell death. Similarly, there was no light-dose dependent response in the absence of ALA.

### Clonogenic cell survival following γ-irradiation

The radiation dose–survival curves for the four cell lines are shown in Figure 3. The data shown are from pooled experiments ($n \geq 3$). The data were fitted by a linear quadratic function for each cell line by means of least squares Marquardt algorithm. Cell line characteristics and survival curve parameters are shown in Table 1. In order to compare the in vitro radiosensitivity of the cell lines we used the surviving fraction following treatment with 2 Gy of ionizing radiation (SF2) in accordance with previous studies (Brock et al, 1990; Girinsky et al, 1994; Champion et al, 1997). The SF-2 value was derived by solving the equation $SF2 = e(-(\alpha D + \beta D^2))$ for D = 2 Gy. A fairly wide variation in radiation sensitivity was observed in these cell lines, with SF2 values of 0.37 (SCC61), 0.61 (V175), 0.54 (V134), and 0.35 (VB001).

### DNA cell cycle kinetics following photodynamic therapy or γ-irradiation

Histograms of cellular DNA content following various treatments are given in Figure 4. It can be seen that following 8 Gy of radiation the three tumour cell lines (V175, V134, SCC61) exhibit an accumulation of cells in the G2 phase of the cell cycle, whereas the normal fibroblasts (VB001) accumulate in both G1 and G2. This

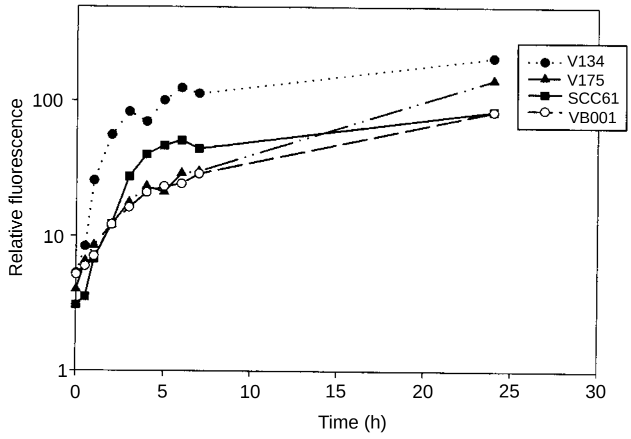

**Figure 1** Fluorescence kinetics of ALA-induced PpIX in the three head and neck cell lines and the normal fibroblasts used in this study. Data points represent the geometric mean of 5 × $10^3$ observations of cellular fluorescence

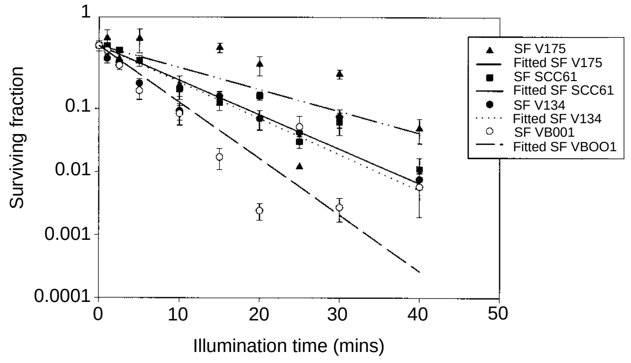

**Figure 2** Clonogenic cell survival curves for the four cell lines used in this study, following exposure to 5-ALA-mediated photodynamic therapy. Data were fitted by a linear quadratic function. In this figure and all subsequent figures, the error bars represent one standard error of the mean and are shown if greater than the symbol

© 2000 Cancer Research Campaign

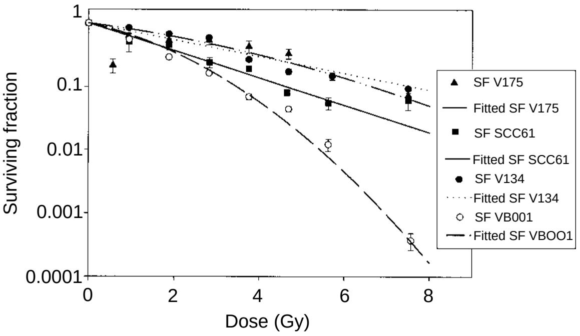

**Figure 3** Clonogenic cell survival curves for the four cell lines following exposure to γ-irradiation observation is consistent with the suggestion that the three tumour cell lines have aberrant p53 function.

The cell line V134 shows a reduced accumulation in G2 following irradiation compared to the other cell lines and this correlates with increased radioresistance of this cell line. For the combination treatment where radiation is given prior to PDT the V134 showed a much reduced accumulation of cells in G2, indicating that cells are recovering from the cell cycle arrest during the 48 hour period between the initial radiation treatment and DNA measurement. PDT on its own had no discernible effect on cell cycle distributions of either tumour or normal cell populations.

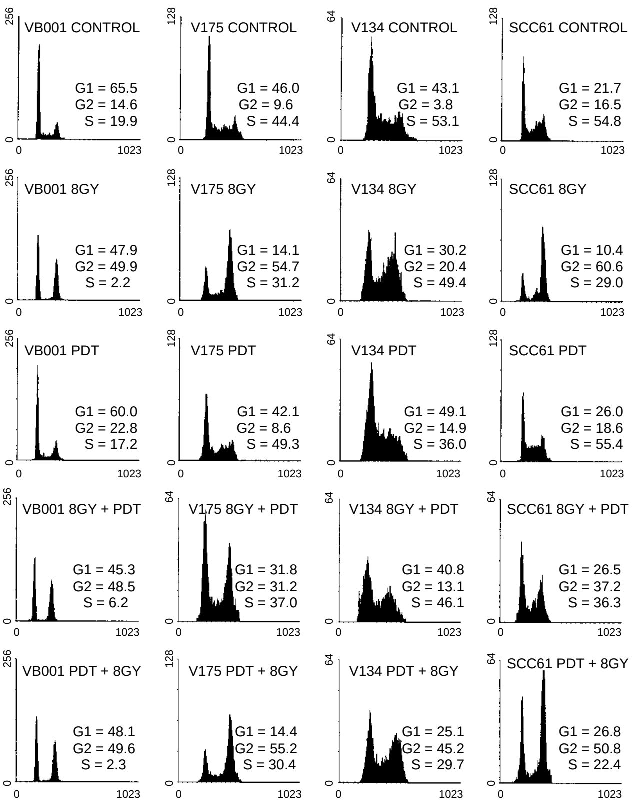

**Figure 4** DNA histograms of the four cell lines used in this study. Cells were exposed to 8 Gy of ionizing radiation or 3 min of PDT or a combination of both treatments. Data represent distributions obtained from 5000 cells

### Clonogenic cell survival following combined PDT and γ-irradiation

In order to investigate the cumulative effects of PDT and γ-irradiation on the cell lines, a fixed 'dose' of PDT was given either 24 hours before, or 24 hours following various doses of γ-irradiation. The light dose used for the PDT was 3 minutes, which from the above data resulted in a surviving fraction in the region of 0.6. The 24 hour delay between treatments was to allow any cell cycle arrest to be maximally in place before the cells underwent the second treatment.

The combined PDT/radiation-dose–survival curves for the four cell lines are shown in Figure 5. The data shown are from pooled experiments ($n \geq 3$). The data were fitted by a linear quadratic function as described above. Also shown on the figure are the theoretical survival curves for each cell line based on the assumption that the combination treatment is purely additive. This curve was obtained by multiplying the radiation dose–surviving fractions by the surviving fraction obtained following 3 min of PDT. It can be seen that none of the cell lines exhibit survival curve parameters which are significantly different from these theoretical curves. The survival curve parameters are summarised in Table 2.

### DISCUSSION

This study has demonstrated the effect of photodynamic therapy used in combination with ionising radiation on clonogenic cell survival of head and neck tumour cell lines. Our results indicate that under the conditions employed in this study the cumulative effects of combined treatments are purely additive.

It has previously been shown that tumour cell sensitivity to PDT varies during the cell cycle (Wyld et al, 1998) with cells in S-phase and G2 being more sensitive. In the case of 5-ALA this is associated with an increased accumulation of PpIX during S-phase and G2. There is also an inverse relationship between PpIX synthesis and cellular iron availability (Rittenhouse-Diakun et al, 1995). S-phase cells may be relatively iron-depleted because of intracellular sequestration of iron by ribonucleotide reductase during DNA synthesis (Brown et al, 1969; Reichard and Ehrenberg 1983) and this may play a role in the increased PpIX synthesis during

**Table 1** Summary of cell characteristics and clonogenic survival data for cell lines treated with ionizing radiation or photodynamic therapy

| Cell line | Doubling time (h)[a] | Clonogenic plating efficiency (% + 1 SE)[a] | Ploidy[a] | γ-radiation SF2 | α(Gy$^{-1}$) (+1SE) | β(Gy$^{-2}$) (+ 1 SE) | PDT SF3 |
|---|---|---|---|---|---|---|---|
| VB001 | nd | 15.1 ± 1.2 | normal diploid | 0.35 | 0.344 ± 0.056 | 0.093 ± 0.009 | 0.54 |
| V134 | 28.8 | 5.67 ± 0.25 | polyploid | 0.55 | 0.304 ± 0.067 | 0.000 ± 0.011 | 0.67 |
| V175 | 35.7 | 2.82 ± 0.23 | hyperdiploid | 0.61 | 0.202 ± 0.051 | 0.022 ± 0.008 | 0.79 |
| SCC-61 | 32.75 | 24.13 ± 1.18 | hyperdiploid | 0.37 | 0.619 ± 0.065 | 0.000 ± 0.011 | 0.68 |

[a]Data from Champion *et al* (1997).

*© 2000 Cancer Research Campaign*

**Table 2** Summary of clonogenic survival data for cell lines treated with ionizing radiation followed by photodynamic therapy and vice-versa

| Cell Line | γ-Radiation + PDT | | | PDT + γ-Radiation | | | Theoretical $SF_2$ |
|---|---|---|---|---|---|---|---|
| | $SF_2$ | $\alpha(Gy^{-1})$ (+1 SE) | $\beta(Gy^{-2})$ (± 1 SE) | $SF_2$ | $\alpha(Gy^{-1})$ (± 1 SE) | $\beta(Gy^{-2})$ (± 1 SE) | |
| VB001 | 0.07 | 0.00 | – | 0.078 | 0.676 ± 0.063 | 0.023 ± 0.012 | 0.114 |
| V134 | 0.25 | 0.00 | – | 0.184 | 0.114 ± 0.091 | 0.046 ± 0.015 | 0.170 |
| V175 | 0.26 | 0.144 ± 0.082 | 0.035 ± 0.014 | 0.327 | 0.00 | 0.060 ± 0.013 | 0.266 |
| SCC-61 | 0.35 | 0.376 ± 0.070 | 0.011 ± 0.012 | 0.496 | 0.314 ± 0.061 | 0.011 ±0.010 | 0.351 |

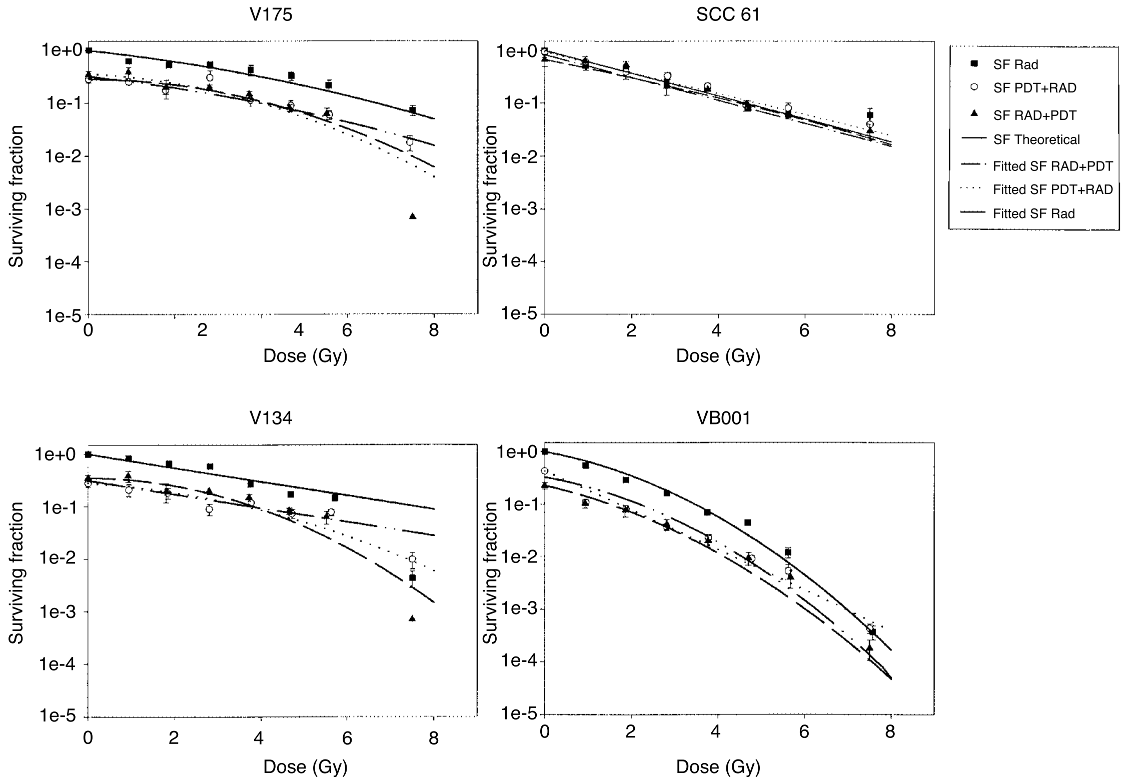

**Figure 5** Clonogenic cell survival curves for the four cell lines. Cells were treated with ionizing radiation, PDT followed 24 h later by ionizing radiation, or radiation followed 24 h later by PDT. Also shown on the figure are the calculated (theoretical) survival curves assuming any combined effect to be additive in nature

S-phase. It has further been observed that rapidly proliferating cells with increased S-phase fractions produce more PpIX and are more PDT-sensitive than slowly proliferating cells (Schick et al, 1995). This relationship between PDT sensitivity and growth rate may also be due to intracellular iron regulation (Pourzand et al, 1999). These marked fluctuations in PDT sensitivity with growth rate may account for the wide scatter observed in our measurements of clonogenic cell survival following PDT.

Mitomycin C has been demonstrated to potentiate photodynamic therapy both at the cellular level (Ma et al, 1992a, 1993a; Datta et al, 1997) and in vivo (Baas et al, 1994, 1996). This potentiation of PDT is associated with a mitomycin-C induced cell cycle arrest in late S-phase/G2 (Ma et al, 1992b). It would therefore be reasonable to expect that a radiation induced cell cycle arrest in G2 would also enhance the effectiveness of PDT. However, we have found no evidence to support this hypothesis and the sensitivity of our cell lines to PDT did not correlate with their relative accumulation of PpIX. Following radiation treatment, the three tumour cell lines used in this study (V175, V134, SCC61) exhibited a cell cycle arrest in G2, whereas the normal fibroblasts arrested in both G1 and G2. This observation is consistent with the suggestion that the three tumour cell lines have aberrant p53 function (Kastan et al, 1991, Kuerbitz et al, 1992), and it is encouraging that this does not appear to be conferring any inherent resistance to the photodynamic treatment.

There have been a number of previous attempts to examine the interaction of PDT and ionizing radiation. However the results presented are conflicting, with some authors presenting evidence of a synergistic interaction (Boegheim et al, 1987; Kostron et al, 1986; Berg et al, 1995), while other investigations show simple additive interaction (Bellnier and Dougherty 1986; Ben-Hur et al, 1988; Schnitzhofer and Krammer 1996). The picture is further complicated by some studies providing evidence of a synergistic effect or an antagonistic effect depending upon the conditions employed within a given system (Ma et al, 1993b, Berg et al, 1995). The causes for the conflicting literature on the combination of radiotherapy and PDT are unclear. Prinsze et al (1992) suggested that cell line differences in the sensitivity to PDT

© 2000 Cancer Research Campaign induced inhibition of DNA repair can explain the conflicting results. Other authors (Kavarnos et al, 1994; Ma et al, 1993b; Berg et al, 1995) suggest that the variations observed in the interaction of PDT and ionizing radiation depend on dose and variation in timing between the two treatments.

In vivo, photodynamic therapy is usually accompanied by severe microvascular changes (Nelson et al, 1987) associated with endothelial damage, microcirculatory stasis, platelet aggregation and haemorrhage, resulting in a coagulation necrosis (Bugelski et al, 1981; Selman et al, 1984; Star et al, 1986; Chaudhuri et al, 1987). These additional effects make it difficult to extrapolate in vitro studies on cell lines to the clinical situation where the combined approach of radiotherapy and photodynamic therapy may have quite different effectiveness than that presented in this study.

In conclusion, head and neck tumour cells were shown to accumulate in G2/M phase of the cell cycle after γ-irradiation. When γ-irradiation was combined with PDT the two treatments acted in an additive manner regardless of the order in which they were given. The interaction between γ-irradiation and PDT may be complicated by dose and timing variations between treatments, and further complicated by fractionated dosing of both the γ-irradiation and PDT. These variable interactions are worthy of further investigation, with a view to defining a role for combined radiotherapy and PDT in tumour therapy.

### REFERENCES

Baas P, Michielsen C, Oppelaar H, van Zandwijk and Stewart FA (1994) Enhancement of interstitial photodynamic therapy by mitomycin C and E09 in a mouse tumour model. *International Journal of Cancer* **56**: 880–885

Baas P, van Geel IPJ, Oppelaar H, Meyer M, Beynen JH, van Zandwijk N and Stewart FA (1996) Enhancement of photodynamic therapy by mitomycin C: a preclinical and clinical study. *British Journal of Cancer* **73**: 945–951

Battle AMC (1993) Porphyrins, porphyrias, cancer and photodynamic therapy – a model for carcinogenesis. *Journal of Photochemistry and Photobiology B Biology* **20**: 5–22

Bellnier DA and Dougherty TJ (1986) Haematoporphyrin derivative photosensitization and gamma-radiation damage interaction in chinese hamster ovary fibroblasts. *International Journal of Radiation Biology* **50**: 659–664

Ben-Hur E, Kol R, Marko R, Riklis E and Rosenthal I (1988). Combined action of phthalocyanine photosensitization and gamma-radiation on mammalian cells. *International Journal of Radiation Biology* **54**: 21–30.

Berg K, Luksiene Z, Moan J and Ma LW (1995). Combined treatment of ionizing radiation and photosensitization by 5-aminolevulinic acid-induced protoporphyrin IX. *Radiation Research* **142**: 340–346

Biel MA (1995). Photodynamic therapy of head and neck cancers. *Seminars in Surgical Oncology* **11**: 355–359

Biel MA (1998). Photodynamic therapy and the treatment of head and neck neoplasia. *Laryngoscope* **108**: 1259–1268

Boegheim JPJ, Dubbelman TMAR, Mullenders LHF and van Steveninck J (1987) Photodynamic effects of haematoporphyrin derivative on DNA-repair in murine L929 fibroblasts. *Biochemical Journal* **244**: 711–715

Brock WA, Baker FL, Wike JL, Sivon SL and Peters LJ (1990). Cellular radiosensitivity of primary head and neck squamous cell carcinomas and local control. *International Journal Of Radiation Oncology Biology-Physics* **18**: 1283–1286

Brown NC, Eliasson R, Reichard P and Thelander L (1969). Spectrum and iron content of protein B2 from ribonucleoside diphosphate reductase. *European Journal of Biochemistry* **9**: 512–518

Bugelski PJ, Porter CW and Dougherty TJ (1981). Autoradiographic distribution of hematoporphyrin derivative in normal and tumout tissue of the mouse. *Cancer Research* **41**: 4604–4612

Champion AR, Hanson JA, Court JB and Venables SE (1995). The micronucleus assay: an evaluation of its use in determining radiosensitivity *in vitro*. *Mutagenesis* **10**: 203–208

Champion AR, Hanson JA, Venables SE, McGregor AD and Gaffney CC (1997). Determination of radiosensitivity in established and primary squamous cell carcinoma cultures using the micronucleus assay. *European Journal of Cancer* **33**(3): 453–462

Chaudhuri K, Keck RW and Selman S (1987). Morphological changes of tumour microvasculature following hematoporphyrin derivative sensitised photodynamic therapy. *Photochemistry and Photobiology* **46**: 823–827

Datta SN, Allmann R, Loh C, Mason M and Matthews PN (1997) Effect of photodynamic therapy in combination with mitomycin C on a mitomycin-resistant bladder cancer cell line. *Br J Cancer* **76**: 312–317

Dougherty TJ (1988) Photodynamic therapy. In *Medical Radiology – Innovations in Radiation Oncology* Withers HR & Peters LJ (eds) pp 175–188. Springer, Berlin.

Dougherty TJ, Cooper MT and Mang TS (1990) Cutaneous phototoxic occurrences in patients receiving Photofrin. *Lasers in Surgery and Medicine* **10**: 485–488

Feyh J (1996) Photodynamic treatment for cancers of the head and neck. *Journal of Photochemistry and Photobiology B: Biology* **36**: 175–177.

Girinsky T, Bernheim A, Lubin R, Tavakol-Razavi T, Baker F, Janot F, Wibault P, Cosset JM, Dwillard P, Duverger A and Fertil B (1994) In vitro parameters and treatment outcome in head and neck cancers treated with surgery and/or radiation: cell characterisation and correlations with local control and overall survival. *International Journal of Radiation Oncology Biology-Physics* **30**: 789–794.

Kastan MB, Zhan Q, el-Deiry WS, Carrier F, Jacks T, Walsh WV, Plunkett BS, Vogelstein B and Fornace AJ (1992) A mammalian cell cycle checkpoint pathway utilizing p53 and GADD45 is defective in ataxia-telangiectasia. *Cell* **71**: 587–597

Kavarnos G, Nath R and Bongiorni P (1994) Visible-light and X irradiation of chinese hamster lung cells treated with hematoporphyrin derivative. *Radiation Research* **137**: 196–201.

Kennedy JC and Pottier RH (1994) Using δ-aminolevulinic acid in cancer therapy. In: *Porphyric Pesticides: Chemistry, Toxicology and Pharmaceutical Applications*, Duke SO and Rebeiz CA (Eds). ACS Symposium Series No 559 pp 291–301. American Chemical Society, Washington

Kennedy JC, Pottier RH and Pross DC (1991) Medical applications of selective tissue photosensitization induced by exogenous 5-aminolevulinic acid. *Photochemistry and Photobiology* **53** (supp): 100S.

Kostron H, Swartz MR, Miller DC Martuza RL (1986) The interaction of hematoporphyrin derivative, light and ionizing radiation in a rat glioma model. *Cancer* **57**: 964–970.

Kuerbitz SJ, Plunkett BS, Walsh WV and Kastam MB (1992) Wild-type p53 is a cell cycle checkpoint determinant following irradiation. *Proc Natl Acad Sci USA* **89**: 7491–7495

Loh CS, Vernon D, MacRobert AJ, Bedwell T, Bown SG and Brown SB (1993) Endogenous porphyrin distribution induced by 5-aminolevulinic acid in the tissue layers of the gastrointestinal tract. *Journal of Photochemistry and Photobiology B: Biology* **20**: 47–54

Ma LW, Moan J, Steen HB, Berg K and Peng Q (1992a) Effect of mitomycin C on the uptake of photofrin II in a human adenocarcinoma cell line. *Cancer Letters* **64**: 155–162

Ma LW, Steen HB, Moan J, Berg K, Peng Q, Saether H Rimington C (1992b) Cytotoxicity and cytokinetic effects of mitomycin C and/or photochemotherapy in a human colon adenocarcinoma cell line. *International Journal of Biochemistry* **24**: 1807–1813

Ma LW, Moan J, Berg K, Peng Q and Steen HB (1993a) Potentiation of photodynamic therapy by mitomycin C in cultured human colon adenocarcinoma cells. *Radiation Research* **134**: 22–28

Ma LW, Iani V and Moan J (1993b) Combination therapy: photochemotherapy; electric current; and ionizing radiation. Different combinations studied in a WiDr human colon adenocarcinoma cell line. *Journal of Photochemistry and Photobiology* **21**: 149–154.

Nash JC (1979) *Compact numerical methods for computers; linear algebra and function minimisation.* Adam Hilger, Bristol.

Nelson JS, Liaw LH and Berns MW (1987) Tumour destruction in photodynamic therapy. *Photochemistry and Photobiology* **46**: 829–835.

Ormerod MG, Payne AWR and Watson JV (1987) Improved program for the analysis of DNA histograms. *Cytometry* **8**: 637–641.

Peng Q, Warloe T, Berg K, Moan J, Kongshaug M, Giercksky KE and Nesland JM (1997) 5-Aminolevulinic acid based photodynamic therapy; clinical research and future challenges. *Cancer* **79**(12): 2282–2308.

Pourzand C, Reelfs O, Kvam E and Tyrell RM (1999) The iron regulatory protein can determine the effectiveness of 5-aminolevulinic acid in inducing protoporphyrin IX in human primary skin fibroblasts. *Journal of Investigative Dermatology* **112**(4): 419–425

Prinsze C, Penning LC, Dubbelman TMAR and van Steveninck J (1992) Interaction of photodynamic treatment and either hyperthermia or ionizing radiation and of

 *© 2000 Cancer Research Campaign*

ionizing radiation and hyperthermia with respect to cell killing of L929 fibroblasts, chinese hamster ovary cells, and T24 human bladder cells. *Cancer Research* **52**: 117–120

Reichard P and Ehrenberg A (1983) Ribonucleotide reductase – a radical enzyme. *Science* **221**: 514–518.

Rittenhouse-Diakun K, van Leengoed H, Morgan J, Hryhorenko E, Paszkiewicz G, Whitaker JE and Oseroff AR (1995) The role of transferrin receptor (CD71) in photodynamic therapy of activated and malignant lymphocytes using the haem precursor δ-aminolaevulinic acid. *Photochemistry and Photobiology* **61**: 523–528.

Schick E, Kaufman R, Ruck A, Hainzl A and Boehncke WH (1995) Influence of activation and differentiation of cells on the effectiveness of photodynamic therapy. *Acta Dermato Venereologica* **75**: 276–279.

Schnitzhofer GM and Krammer B (1996) Photodynamic treatment and radiotherpay combined effect on the colony-forming ability of V79 Chinese hamster fibroblasts. *Cancer Lett* **108**: 93–99

Schuller DE, McCaughan JS and Rock RP (1984) Photodynamic therapy in head and neck cancer. *Archives of Otolaryngology* **111**: 351–355.

Selman SH, Kreimar-Birnbaum M, Klaunig JE, Goldblatt PJ, Keck RW and Britton SL (1984) Blood flow in transplantable bladder tumours treated with haematoporphyrin derivative and light. *Cancer Research* **44**: 1924–1927

Star WM, Marjnissen HPA, van den Berg-Blok AE, Versteeg JA, Franken KA and Reinhold HS (1986) Destruction of rat mammary tumour and normal microcirculation by hematoporphyrin derivative photoradiation observed in vivo in sandwich observation chambers. *Cancer Research* **46**: 2532–2540

van Dongen GA and Snow GB (1997) Prospects for future studies in head and neck cancer. *European Journal of Surgical Oncology* **23**(6): 486–491

Watson JV, Chambers SH and Smith PJ (1987) A pragmatic approach to the analysis of DNA histograms with a definable G1 peak. *Cytometry* **8**: 1–8

Weichselbaum RR, Dahleberg W, Little JB, Ervin TJ, Miller D and Hellman S (1984) Cellular x-ray repair parameters of early passage squamous cell carcinoma lines derived from patients with known responses to radiotherapy. *British Journal of Cancer* **49**: 595–601

Weichselbaum RR, Beckett MA, Schwartz JL and Dritschilo A (1988) Radioresistant tumour cells are present in head and neck carcinomas that recur after radiotherapy. *International Journal of Radiation Oncology Biology Physics* **15**: 575–579

Wile AG, Novotny J and Mason GR (1984) Photoradiation therapy of head and neck cancer. In *Porphyrin Localisation and Treatment of Tumours.* Doiron DR & Gomer CJ (eds), pp 681–693. AR Liss, New York.

Wyld L, Smith O, Lawry J, Reed MWR and Brown NJ (1998) Cell cycle phase influences tumour cell sensitivity to aminolaevulinic acid-induced photodynamic therapy in vitro. *British Journal of Cancer* **78**: 50–55.

*© 2000 Cancer Research Campaign*
